# Attitude Toward Seeking Professional Psychological Help Among Community-Dwelling Population in China

**DOI:** 10.3389/fpsyt.2020.00417

**Published:** 2020-05-14

**Authors:** Pan Chen, Xiu Jun Liu, Xiao Qin Wang, Bing Xiang Yang, Juan Ruan, Zhongchun Liu

**Affiliations:** ^1^School of Health Sciences, Wuhan University, Wuhan, China; ^2^Affiliated Mental Health Center, Tongji Medical College of Huazhong, University of Science & Technology, Wuhan, China; ^3^Department of Psychiatry, Renmin Hospital of Wuhan University, Wuhan, China

**Keywords:** attitude, Chinese, community-dwelling population, help-seeking behaviour, professional psychological help

## Abstract

**Objective:**

To explore the attitudes and factors in seeking professional psychological help among a Chinese community-dwelling population in order to promote positive help-seeking behaviors and better utilization of mental health services.

**Methods:**

Using system and simple random sampling with Kish selection table methods, 912 community-dwelling residents were included in this study and asked about their attitudes toward seeking professional psychological help, depression symptoms, family function, depression literacy, help-seeking intention, and stigma.

**Results:**

Scores on the Attitudes Toward Seeking Professional Psychological Help scale (ATSPPH-SF) indicated a neutral attitude toward openness to seeking treatment for psychological problems and a negative attitude toward the value and need to seek treatment with a negative total score. Multiple linear regression analysis showed that gender, age, social support (employment status and family function), depression literacy, stigma, and help-seeking intention are significantly associated with attitude toward seeking professional psychological help.

**Conclusion:**

The overall attitude toward seeking professional psychological help is not optimistic, thus, more efforts are needed to enhance understanding. Effective interventions including mental health education, training of mental health professionals, and popularizing the use of mental health services are essential, especially for the at-risk population.

## Introduction

Mental disorders have become a serious problem worldwide, with over 300 million (4.4%) and 264 million (3.6%) people experiencing depression and anxiety disorders respectively (1). A recent large-scale survey in China showed that the lifetime prevalence of depression and anxiety was 6.9 and 7.6%, respectively (2). Mental disorders can cause high rates of disability and mortality as well as increase the burdens of other diseases and related health care costs (3–5) and suicide is the most serious consequence (6, 7). A majority of individuals (76–85%) with mental disorders receive no treatment in low- and middle-income countries compared with 35–50% in high-income countries (8). A low rate of help-seeking behaviors (9–11) and delayed help-seeking (12) are barriers to receiving timely and effective treatment for persons with mental disorders. Professional help-seeking (e.g. from mental health professionals, physicians) is more effective in the prevention and management of mental health problems and protects individuals against suicide (13, 14).

Currently, the outlook is not optimistic regarding the use of professional help-seeking behaviors for mental health issues in many countries, especially China (15, 16). In Canada, one in 10 young people had consulted professionals for their problems (17). In another study across four Chinese provinces, it was found that only 11 (7.3%) of the suicide decedents (151) and four (3.3%) of those who attempted (120) had previously sought psychological or medical treatment for psychological problems (18). A recent study indicated that 7.9% of participants with depressive symptoms had sought help from mental health professionals (MHPs) and 3.7% from general physicians (19).

Based on the theory of planned behavior (TPB) (20), attitude can indirectly influence help-seeking behavior *via* intention (21), and it is a precursor to help-seeking intention and actual help behaviors (22). Thus, improving help-seeking attitude is the first step in promoting further help-seeking behaviors.

Attitudes toward seeking professional psychological help (ATSPPH) vary by geographic location. The National Comorbidity Survey (NCS) of the USA conducted in 1990–1992 showed that one-third of respondents would definitely seek professional help (23). A study in Saudi Arabia (24) noted that 43.5% of participants would use professional assistance for serious emotional problems. In Europe, over 69% of survey participants had a more open attitude toward seeking professional help and approximately 50% recognized the value of professional help (25). In the Western Pacific region, participants had a poor attitude compared with results in the US and Europe (26). A 40-year review from 1968 to 2008 showed that the ATSPPH has become increasingly negative over time (27). In China, most research about help-seeking attitude has focused on college students (28–30). One study found that 40.4% of Chinese university students expressed willingness to seek help from a psychiatrist if they had suicidal ideation. And they preferred to seek informal social networks rather than professionals to solve their mental health issues (31). Currently, research about ATSPPH in the Chinese community-dwelling population is lacking.

Attitudes toward seeking professional psychological help can be affected by various factors such as gender (32), age (25), educational level (33), marital status (34), work status (35); sociological and cultural factors such as culture prejudice (36), social support (37), mental health literacy (38); individual factors such as stigma related to mental problems (39), help-seeking intention (40), the experience of mental problems (17), knowledge about the role of health professionals (41), and personality traits (42). Based on the above findings, this study aimed to further explore significant correlates in the Chinese community-dwelling population which have been neglected in previous studies.

## Materials and Methods

### Sample Size and Sample Design

This study was a population-based, cross-sectional survey conducted between January 2017 and December 2017 in Wuhan, a major city in central China (43). A stratified random sampling method was applied in the whole sampling process, which was divided into three stages to select target communities, households, and individuals. First, 20 communities from seven central districts of Wuhan were selected using an Excel generate random number table. Second, a systematic sampling method was used by coding all households in each community with a random number from an Excel generated random number table, and identifying 50 target households in each community according to the sampling interval K (the total number of households in the community divided by 50). Finally, in Kish selection table method, one of eight codes (A, B1, B2, C, D, E1, E2, F) was assigned to the target households; a family registration form (including name, age, gender, member number) was used to assign additional codes to individual family members. This allowed for the use of a Kish code for every household to determine the target individuals. This Kish selection table method is usually used to determine target individuals from every family in a household survey (44). A total of 1,000 questionnaires were distributed; 923 questionnaires were returned (response rate: 92.3%) and 912 questionnaires were valid (effective callback rate: 91.2%). Inclusion criteria were: minimum age of 15 years and able to read and write in Mandarin. Participants with severe somatic illness or other psychosis and related disorders, dementia, and mental impairment due to substance dependence were excluded.

Institutional Review Board of Wuhan University School of Medicine granted approval for this study. Based on the principle of voluntary participation and withdrawal from the study at any time, participants signed informed consent prior to participation. Privacy rights were protected by using coded anonymous questionnaires and the security of these questionnaires was maintained. Small gifts were provided in gratitude for an individual’s participation.

### Measures

#### Socio-Demographic Characteristics

Participants completed a general demographic questionnaire indicating their gender, age, religion, education level, parents’ and spouse’s education level, employment, and marital status.

#### Attitudes Toward Seeking Professional Psychological Help Scale—Short Form (ATSPPH-SF)

The ATSPPH-SF was developed by Fischer and Farina (45), translated into Chinese in 2017 and revised for cultural applicability after communicating with the original authors. The original questionnaire was translated by two individuals, an assistant professor in mental health nursing with a doctoral degree and having expertise in mental health and concepts used in the scale and a doctoral student majoring in teaching the Chinese language. The researchers then compared and synthesized the differences and modifications in the two translations to develop a preliminary version of the scale. It was submitted for review to two experts who have been engaged in mental health research for 10 years. In addition, two individuals with no experience using the scale were invited separately to do back-translations. One was a nursing professor who is bilingual and has worked in an English-speaking country for at least 10 years and the other was a master’s student in English. The researchers compared the back-translation versions with the original scale, discussed and reviewed it with four translators to develop a Chinese version of the original scale.

The scale was used to measure participants’ ATSPPH and included two dimensions: openness to seeking professional help for emotional problems (*items 1, 3, 5, 6, 7*) with item scores ranging from zero (disagree) to three (agree); value and need in seeking professional help (*items 2, 4, 8, 9, 10*) with items scored in reverse (zero = agree and three = disagree). The total score of the scale ranges from zero to 30 with higher scores indicating a better help-seeking attitude. The cut-off score on the scale is greater than 20 points and for each dimension is greater than 10 points; otherwise, the attitude is deemed to be negative.

In the original study of college students, the short version of the scale demonstrated internal consistency ranging from 0.82 to 0.84, a one-month test–retest reliability of 0.80; and, a correlation of 0.87 with the longer scale (45). It had good validity and could distinguish whether they were willing to seek professional psychological help (46). In the current study, internal consistency was shown with a Cronbach’s α for the entire scale and the two subscales of 0.681, 0.714 and 0.657, respectively. The test-retest reliability was 0.895 for the entire scale, 0.781 for the openness scale and 0.827 for the value and need scale. The Chinese version of the ATSPPH-SF is suggested to be valid and reliable. The item content validity (I-CVI) was 0.833–1.000, and the scale content validity index (S-CVI) was 0.932. The result of exploratory factor analysis showed that the cumulative contribution rate of the two common factors was 45.346%, the correlation coefficient between each dimension and the total score of the scale was 0.755–0.772, and the correlation coefficient of the two dimensions was 0.167, both of which were statistically significant (*P* < 0.01). The KMO value was 0.783, Bartlett’s test χ2 = 1505.611, P < 0.01. Confirmatory factor analysis showed that each indicator fit well (χ2/df = 2.53, RMR = 0.035, RMSEA = 0.054, NFI = 0.927, CFI = 0.938) (47). Thus, the use of a two-factor analysis model in this study was found to be robust.

#### Centre for Epidemiological Studies Depression Scale (CES-D)

The CES-D scale (48) has 20 items with a four-point Likert scale ranging from 0 (no or hardly) to 3 (almost always) (49), which is used to screen for participants’ weekly depressive symptoms. The total score ranges from zero to 60 with higher scores indicating greater depressive symptoms. In the current study, the Cronbach’s α was 0.873.

#### Family APGAR Index

To measure an individual’s satisfaction with family functioning, the current study used the Family APGAR Index developed by Smilkstein (50). It consists of five items standing for Adaptation, Partnership, Growth, Affection, and Resolution and uses a three-point Likert scoring from 0 (hardly ever) to 2 (almost always). The total score ranges from zero to 10 with higher scores indicating better family function. Good reliability was shown in this study with a Cronbach’s α of 0.889.

#### Depression Stigma Scale (DSS)

This scale was used to measure personal and public stigma toward depression (51) and had good reliability with a Cronbach’s α of 0.814. It consists of 18 items with two subscales: personal stigma (personal attitude on depression) and public stigma (attitudes toward others’ views on depression). Each subscale includes nine items using a five-point Likert scoring, which ranges from 4 (strongly agree) to 0 (strongly disagree). Total scores on each of the two subscales ranges from zero to 36 points with a higher score representing increased personal and public stigma.

#### Depression Specific Self-Management (DSSM) Scale

To measure the depressive knowledge of a community-dwelling population, the current study selected the first dimension of this scale: specific knowledge of depression (items one to four) (52). This scale uses a five-point Likert scoring with a higher score indicating a higher level of depression knowledge. The Cronbach’s α was 0.542 in the current study.

#### General Help-Seeking Questionnaire (GHSQ)

The community residents’ help-seeking intention for psychological problems was measured by the GHSQ which includes informal and professional help-seeking sources (53). The latter was used to measure the professional help-seeking intention. It uses a seven-point Likert scale ranging from 1 (extremely unlikely) to 7 (extremely likely) with higher scores indicating greater help-seeking intention. Reliability was good with a Cronbach’s α of 0.618.

### Statistical Analysis

Data analysis was conducted using SPSS 22.0. Descriptive statistics were used to analyze variables. Frequency, means, and standard deviations were used to describe participants’ characteristics and ATSPPH-SF scores. To better understand the difference in the ATSPPH between China and other different regions, independent sample t-tests were performed. Independent sample *t*-tests and one-way ANOVA tests were conducted to assess ATSPPH-SF scores with categorical variables. Pearson correlation analysis was to explore the relationship between continuous variables (e.g. age, family function, stigma, help-seeking intention, depressive symptoms, and depression knowledge) and help-seeking attitude. Variables that were statistically significant (*P* < 0.05) based on a one-way ANOVA test, independent sample t-test, and Pearson correlation analysis were entered into a multiple linear regression model, which was used to explore the significant correlates of help-seeking attitude. The multicollinearity was tested using SPSS when conducting the multiple linear regression analyses, which confirmed that there were no issues of multicollinearity (VIF ranged from 1.016 to 1.670). The pattern of missing data per variable was missing completely at random (Little’s MACR test: Chi-square = 51.947, df = 44, *P* = 0.192). Respondents with four or more missing values on the included variables were excluded. When a questionnaire lacked ≤4 item responses, the mean score of non-missing items was used in place of the scores of the missing items. Results were considered statistically significant with a level of *P* < 0.05.

## Results

### Sample Characteristics

There were 912 participants included: 255 were male (27.9%) and 657 were female (72.1%). The average age was 38.66 ± 17.34 years (see **Table 1**). In addition, the score of the Family APGAR Index ranged from 0 to 10 (Mean = 7.20, SD = 3.01), indicating participants had good family function on average. The score of personal stigma ranged from 2 to 36 (M = 19.22, SD = 5.04) and public stigma ranged from 5 to 36 (M = 21.94, SD = 5.02). The score of depression knowledge ranged from 4 to 20 (M = 14.14, SD = 2.35) and the score of help-seeking intention from professionals ranged from 9 to 63 (M = 22.09, SD = 13.97).

**Table 1:** Socio-demographic characteristics of the study sample (N = 912).

Characteristics	n	%
Gender (*n* = 912)		
Male	255	28.0
Female	657	72.0
Religious affiliation (*n* = 909)		
No	865	95.2
Yes	44	4.8
Education level (*n* = 911)		
Less than high school	176	19.3
Junior high school/high school/some college	402	44.1
Bachelor’s degree or higher	333	36.6
Father’s education level (*n* = 889)		
Less than high school	468	52.6
Junior high school/high school/some college	329	37.0
Bachelor’s degree or higher	92	10.3
Mother’s education level (*n* = 887)		
Less than high school	565	63.7
Junior high school/high school/some college	263	29.7
Bachelor’s degree or higher	59	6.6
Spouse’s education level (*n* = 627)		
Less than high school	171	27.3
Junior high school/high school/some college	269	42.9
Bachelor’s degree or higher	187	29.8
Employment status (*n* = 909)		
Unemployed/laid-off/retired	295	32.5
Full time/part time	614	67.5
Employment type (*n* = 860)		
Skilled worker/farmer/business man/other	225	26.2
General company/state-owned enterprise or public institution staff/civil servant	483	56.2
Students	152	17.7
Marital status (*n* = 907)		
Single/separated/divorced/widowed	319	35.2
Cohabiting/married/remarried	588	64.8

### Scores of the ATSPPH-SF

The total score on the ATSPPH-SF was less than 20 (*M* = 18.13, *SD* = 5.63) indicating that overall attitude was negative. The score on openness was near the critical value of 10 (*M* = 10.05, *SD* = 3.74) indicating a neutral attitude and the score on value and need was less than 10 points (*M* = 8.09, *SD* = 3.53) indicating a negative attitude. Participants were in greatest agreement on the third item “*If I were experiencing a serious emotional crisis at this point in my life, I would be confident that I could find relief in psychotherapy*” having the highest score (*M* = 2.32*, SD* = 0.93*)* and with the ninth item “*Each person should solve their own problem and not seek psychological consultation unless absolutely necessary”* having the lowest score (*M* = 1.46*, SD* = 1.14) on reverse scoring. Items of least agreement among the participants were the sixth item “*I might want to have psychological counseling in the future*” (*M* = 1.60*, SD* = 1.16) and the second item “*The idea of talking about problems with a psychologist strikes me as a poor way to get rid of emotional conflicts*” (*M* = 2.23*, SD* = 1.02) (see **Table 2**).

**Table 2:** Scores on the attitude toward seeking professional psychological help scale-short form (ATSPPH-SF).

Items	*M*	*SD*	*Min*	*Max*
1. If I believed I was having a mental breakdown, my first inclination would be to get professional attention.	2.05	1.12	0.00	4.00
*2. The idea of talking about problems with a psychologist strikes me as a poor way to get rid of emotional conflicts.	2.23	1.02	0.00	3.00
3. If I were experiencing a serious emotional crisis at this point in my life, I would be confident that I could find relief in psychotherapy.	2.32	0.93	0.00	3.00
*4. There is something to admire about a person who copes with conflicts and fears without going for professional help.	1.46	1.14	0.00	3.00
5. I would want to get psychological help if I was worried or upset for a long period of time.	2.17	1.03	0.00	4.00
6. I might want to have psychological counseling in the future.	1.60	1.16	0.00	4.00
7. A person with an emotional problem is not likely to solve it alone; he or she is likely to solve it with professional help.	1.91	1.01	0.00	3.00
*8. Considering the time and expense involved in psychotherapy, it would have little value for a person like me.	1.72	1.07	0.00	3.00
*9. A person should work out his or her own problems; getting psychological counseling would be a last resort.	1.16	1.11	0.00	3.00
*10. Personal and emotional troubles, like many things, tend to work out by themselves.	1.51	1.16	0.00	3.00
Total score	18.13	5.64	0.00	30.00
Openness to seeking treatment for emotional problems	10.05	3.74	0.00	15.00
Value and need in seeking treatment	8.09	3.53	0.00	15.00

Openness to seeking professional psychological help for emotional problems (item #1, 3, 5, 6, 7); (0 = disagree, 1 = partially disagree, 2 = partially agree, 3 = agree).

*Value and need in seeking professional psychological help (item # 2, 4, 8, 9, 10); (0 = agree, 1 = partially agree, 2 = partially disagree, 3 = disagree).

M, mean; SD, standard deviation; Min, Minimum score; Max, Maximum score.

An investigation of ATSPPH in western Pacific regions showed that a lower score was found in the Philippines (*M* = 16.83, *SD* = 4.13, *t* = 2.759, *P* = 0.006), but there were no significant differences in Fiji (*M* = 18.47, *SD* = 7.76, *t* = −0.644, *P* = 0.520) and Cambodia (*M* = 18.69, *SD* = 3.47, *t* = −1.182, *P* = 0.238) (26).There were also lower average scores (*M* = 17.4, *SD* = 5.5, *t* = 3.602, *P* < 0.001) in Europe including Germany (*M* = 17.2, *SD* = 4.4, *t* = 4.405, *P* < 0.001), Hungary (*M* = 13.9, *SD* = 4.3, *t* = 18.555, *P* < 0.001), Ireland (*M* = 18.4, *SD* = 5.4, t = −1.070, *P* < 0.001), and Portugal (*M* = 20.0, *SD* = 5.7, *t* = −7.222, *P* < 0.001) which had a higher score than the results of this study (*M* = 18.13, *SD* = 5.63) (25). Higher scores were also found in the USA (*M* = 20.45, *SD* = 5.51, *t* = −5.226, *P* < 0.001) (54).

### Differences in the ATSPPH Scores According to Participants’ Socio-Demographic Characteristics

The results of ANOVA-test and *t*-test indicated that there were significant differences in gender, education level, father’s and spouse’s education level, employment, and marital status related to openness to seeking professional psychological help. Gender, religion, education level, parents’ and spouses’ education level, employment, and marital status had statistically significant differences related to value and need in seeking professional help (see **Table 3**).

**Table 3:** Comparison of the ATSPPH-SF scores by socio-demographic characteristics.

Characteristics	Openness to seeking professional help				Value and need in seeking professional help			
	M(SD)	*t*	*F*	*P-*value	*M (SD)*	*t*	*F*	*P-*value
**Gender (*n* = 912)**		−2.451		0.015		2.211		0.027
Male	9.56(3.71)				8.49(3.39)			
Female	10.23(3.74)				7.93(3.58)			
**Religion affiliation (n = 909)**		0.190		0.850		2.617		0.009
No	10.04(3.69)				8.15(3.48)			
Yes	9.93(4.71)				6.73(4.24)			
**Education level (n = 911)**			7.579	0.001			21.144	<0.001
Less than high school	9.06(4.91)				6.68(3.84)			
Junior high school/high school/some college	10.25(3.51)				8.1(3.51)			
Bachelor’s degree or higher	10.31(3.18)				8.77(3.14)			
**Father’s education level (n = 889)**			3.809	0.023			8.815	<0.001
Less than high school	9.83(4.03)				7.71(3.64)			
Junior high school/high school/some college	10.55(3.27)				8.76(3.30)			
Bachelor’s degree or higher	9.92(2.99)				8.21(3.01)			
**Mother’s education level (*n* = 887)**			0.949	0.387			8.475	<0.001
Less than high school	10.02(3.90)				7.77(3.59)			
Junior high school/high school/some college	10.40(3.27)				8.80(3.17)			
Bachelor’s degree or higher	10.05(3.01)				8.56(3.34)			
**Spouse’s education level (*n* = 627)**			5.287	0.005			10.706	<0.001
Less than high school	9.27(4.78)				6.62(3.70)			
Junior high school/high school/some college	10.28(3.84)				7.88(3.62)			
Bachelor’s degree or higher	10.58(3.44)				8.29(3.39)			
**Employment status (n = 909)**		−4.051		<0.001		-7.521		<0.001
Unemployed/laid-off/retired	9.33(4.59)				6.85(3.62)			
Full time/part time	10.40(3.20)				8.68(3.34)			
**Employment type (*n* = 860)**			2.135	0.119			15.274	<0.001
Skilled worker/farmer/business man/other	9.71(4.51)				7.57(3.84)			
General company/state-owned enterprise or public institution staff/civil servant	10.24(3.68)				7.91(3.48)			
Students	9.70(2.80)				9.48(2.89)			
**Marital status (*n* = 907)**		−1.165		0.244		4.105		<0.001
Single/separated/divorced/widowed	9.86(3.30)				8.71(3.25)			
Cohabiting/married/remarried	10.15(3.96)				7.74(3.64)			

M, mean; SD, standard deviation; t, independent sample t-test; F, ANOVA-test ATSPPH-SF, attitude toward seeking professional psychological help scale-short form.

### Pearson Correlation Analysis Between ATSPPH and Continuous Variables

**Table 4** shows that age, family function, depression literacy, and help-seeking intention positively correlated to “openness” to help-seeking. Of these, age, depression literacy, and help-seeking intention were positively correlated to “value and need” of professional psychological help. In addition, personal stigma was negatively related to help-seeking attitude on “openness” and “value and need.” Public stigma was also negatively related to help-seeking attitude on “value and need” (see **Table 4**).

**Table 4:** Correlation between age, help-seeking intention, stigma, depressive symptoms, depression literacy, family function, and ATSPPH.

Variables	Openness to seeking professional help		Value and need in seeking professional help	
	Correlation coefficient(r)	*P*-value	correlation coefficient(r)	*P*-value
Age	−0.081	0.014	−0.306	<0.001
Help-seeking intention	0.278	<0.001	0.198	<0.001
Personal stigma	−0.119	<0.001	−0.304	<0.001
Public stigma	−0.042	0.208	−0.222	<0.001
Depression symptoms	0.019	0.570	−0.029	0.389
Depression literacy	0.203	<0.001	0.079	0.018
Family function	0.103	0.002	0.031	0.356

### Influencing Factors on the ATSPPH

Two multiple linear regression models were both statistically significant, F *_openness_* = 30.616, *P* < 0.001, with a R-square value (R^2^) of 0.149; F *_value__and__need_* = 39.412, *P* < 0.001, R^2^ = 0.153. Results showed that participants who were female (Beta = 0.079; 95%CI: 0.140, 1.172), were employed (Beta = 0.112; 95%CI: 0.400, 1.389), had better family function (Beta = 0.068; 95%CI: 0.007, 0.160), as well as increased help-seeking intention (Beta = 0.288; 95%CI: 0.060, 0.093) and depression literacy (Beta = 0.156; 95%CI: 0.150, 0.348) were significantly associated with higher scores in openness to seeking professional help for emotional problems. Older participants (Beta = −0.208; 95%CI: −0.056, −0.029), those having personal (Beta = −0.150; 95%CI: −0.159, −0.049) and public stigma (Beta = −0.103; 95%CI: −0.123, −0.021) were significantly associated with lower “value and need in seeking professional help,” while those with increased help-seeking intention (Beta = 0.125; 95%CI: 0.016, 0.047) had a more positive attitude (see **Table 5**).

**Table 5:** Correlates of attitudes toward seeking professional psychological help (ATSPPH) conducted by multiple linear regression.

Variables	Openness to seeking professional help						Value and need in seeking professional help			
	B(SE)	Beta	*P-*value	95%CI			B(SE)	Beta	*P-*value	95%CI
Gender (Male = 0, Female = 1)	0.656(0.263)	0.079	0.013	0.140 1.172			–	–	–	–
Employment status (No = 0, Yes = 1)	0.894(0.252)	0.112	<0.001	0.400 1.389			–	–	–	–
Age	–	–	–	–			−0.042(0.007)	−0.208	<0.001	−0.056 −.029
Help-seeking intention	0.077(0.008)	0.288	<0.001	0.060 0.093			0.031(0.008)	0.125	<0.001	0.016 0.047
Family function	0.083(0.039)	0.068	0.033	0.007 0.160			–	–	–	–
Personal stigma	–	–	–	–			−0.104(0.028)	−0.150	<0.001	−0.159 −0.049
Public stigma	–	–	–	–			−0.072(0.026)	−0.103	0.006	−0.123 −0.021
Depression literacy	0.249(0.050)	0.156	<0.001	0.150 0.348			–	–	–	–

“Stepwise” method used in the multivariate models.

B, unstandardized coefficients; SE, standard error; Beta, standardized coefficients.

Employment status: No: Unemployed/laid-off/retired; Yes: Full/part time.

## Discussion

### ATSPPH

The ATSPPH was negative in this Chinese community-dwelling population (*M* = 18.13, *SD* = 5.63). Compared with the findings in different regions, it can be seen that the ATSPPH in China is more positive than in the Philippines (*M* = 16.83, *SD* = 4.13) (26) and most European regions (*M* = 17.4, *SD* = 5.5) such as Germany (*M* = 17.2, *SD* = 4.4) and Hungary (*M* = 13.9, *SD* = 4.3) (25). However, there are still gaps, compared with the more positive help-seeking attitude in the USA (*M* = 20.45, *SD* = 5.51) (54) and Portugal (*M* = 20.0, *SD* = 5.7) (25), Thus, there is a need to focus on improving understanding of and attitudes regarding professional psychological support in China.

There are several reasons from a cultural, social, and individual point of view to explain this negative help-seeking attitude. Culturally, some Chinese perceive psychotherapy as an inefficient and impractical way of dealing with problems affected by traditional beliefs (55). They may be reluctant to discuss their emotions openly with others to save face (56). In addition, some Chinese believe mental illness is caused by emotional disharmony or by evil spirits (56). People who have cultural prejudice would link mental illness to personal shortcomings and reinforce their stigma (57, 58). Hence, they prefer to solve distress on their own by repressing their emotional problems instead of seeking help from others (32).

Socially, mental health literacy is low in the general population and the spread of psychological education is insufficient (59). For instance, some people regard their psychological distresses as normal and these distresses can go away (60). There is a lack of mental health services in China (61) and the number of qualified community MHPs is very low (62), especially in rural regions. There is evidence that one reason for not seeking help is that members of the community do not know where to go to seek professional help (32).

From the individual’s perspective, their ATSPPH may be affected by the severity and complexity of their psychological problems. People with less severe mental disorders are more likely to choose informal help (11); but unfortunately, those with severe mental disorders do not seek help due to a lack of insight (63). It is suggested that a negative perception of psychological professionals is also a barrier to seeking professional help (10), for instance, being skeptical about the effectiveness of psychological help or the competence of MHPs (64) or having had an unpleasant experience with professionals (63).

### Factors Related to ATSPPH

#### Socio-Demographic Characteristics

Participants in this study had an increasingly negative attitude toward value and need in seeking professional psychological help with increasing age. Older persons may tend to seek help from family or friends rather than professional resources (65). They are rich in life experience and more deeply influenced by Chinese culture (66), making them feel that they have abilities to solve problems and are not willing to seek help. This perspective contrasts with that of younger people who suffer from more mental problems caused by various stresses related to maladaptation, so their demand for professional psychological help is higher than that of older people (67, 68). Moreover, younger people easily obtain more information about mental health from school, the workplace, or the Internet, thus they have a better attitude toward the value of professional help (34).

Consistent with most studies, men had a more negative ATSPPH (32, 33, 69). Masculinity including masculine norms and alexithymia may limit men’s ability to express grief (69). They feel shamed talking about emotional problems (70) and fear damaging the ideal image of the male when seeking help from others (41). In addition, men have alternative solutions to alleviate pain such as alcohol, drugs, and aggressive behaviors (41). These explain why they do not actively seek professional help.

#### Social Support

This study found that people who have good social support (relative to employment status and good family function) have a better ATSPPH. There is evidence that employment status is positively associated with psychological openness (35). Individuals who are employed have more opportunities for communication and interaction with others in their work setting, could solve problems more effectively and have a greater sense of self-worth; thus, they are more open to seek mental health professionals when encountering psychological problems (35). In addition, having a source of income also provides financial support to facilitate help-seeking actions (26).

Good family function had a positive effect on help-seeking attitude. In China, family plays a vital role in providing affective support and psychological comfort (71, 72). Family members can offer advice and share guidelines on problem-solving (73) and decision making (74) and they are always the first source of help-seeking when a family member encounters emotional distress (19, 26, 65).

#### Depressive Literacy

Consistent with previous research, having greater depressive knowledge was significantly associated with a positive attitude toward professional help-seeking (26, 32, 36). People with greater knowledge may diminish the stigma related to mental disorders, and they are more willing to seek professional help according to their need (26, 33, 75).

#### Stigma and Help-Seeking Intention

Consistently, stigma was cited as a negative factor affecting professional psychological help-seeking attitude (10, 76, 77). On one hand, people with personal stigma may hide their thoughts to avoid addressing emotional problems, as they would feel uncomfortable, ashamed, and embarrassed to talk about these with professionals (67). Another issue is related to how the public labels individuals with mental disorders as “sick, neurotic,” so individuals are fearful of being laughed at and discriminated against when seeking professional psychological help (78).

Notably, a significant finding of this study revealed that increased help-seeking intention is positively associated with help-seeking attitude. As previously known, the theory of planned behavior (TPB) confirmed that the more positive an individual’s attitude, the stronger an individual’s intention would be (20). However, a confirmatory study found that changing a person’s intention can result in a change of attitude in turn (40), that is, the greater the intention, the better the attitude.

It is worth noting that the R-square values for the two regression models were both small. However, a low R-square value does not negate the importance of any significant variables (79). The theory of planned behavior (TPB) (20) suggests that attitudes can be affected by behavioral beliefs and attitudes can predict behaviors by indirectly affecting intentions. The theory of knowledge-attitude-practice (KAP) (80) espouses that knowledge can change one’s attitude. Based on these theories, this study explored the relationship between ATSPPH and help-seeking intention, stigma, social support, and depression literacy. And the results showed that these factors were significantly associated with ATSPPH though they might not account for more variance. This provides a reference for further exploration of help-seeking attitudes, intention, and behaviors in future research. Moreover, it can be seen that regression models in psycho-social studies (including the studies of help-seeking attitude) usually had small R-square values. For example, one study about the help-seeking attitude found that the factors accounted for 11.4% of the variance in the multiple regression model (81) and another study had an R-square value of 0.16 (82).

## Limitations

There are several limitations to this research which derived from a large-scale survey. First, this is a cross-sectional survey, so a causal relationship between help-seeking attitude and its influencing factors cannot be drawn. Second, the use of self-rating scales focused on depressive symptoms, personal and public stigma related to depression, and help-seeing intention may result in bias as participants may respond in a socially desirable manner. Third, this study was completed in one city in central China, which limits the generalizability of the findings to other areas of the country. While this study identified statistically significant predictors for the ATSPPH score, the clinical implications and characteristics related to these findings should be considered due to the low beta coefficient values.

## Conclusions and Implications

In general, the Chinese attitude toward seeking professional assistance with mental health issues is not positive. Positive factors affecting this attitude include being female, of a younger age, having social support and help-seeking intention, while the negative factor is stigma. More attention should be paid to vulnerable groups (e.g. older adults, males, and the unemployed).

Based on the current research findings, it is important to consider methods to promote better help-seeking attitudes in the general population by eliminating cultural bias and reducing stigma associated with mental health issues and educating the public about the importance of mental health. The focus of these efforts should be centered on older adults and males. Emphasis should be placed on the use of social media channels to spread relevant information to increase mental health literacy. Government programs focused on mental health education and services should be expanded to close the gap between urban and rural areas and increase accessibility to these programs for the community-dwelling population. In addition, there is a need to strengthen education and training of mental health professionals to promote greater trust between those who seek services and the providers.

## Data Availability Statement

All datasets generated for this study are included in the article.

## Ethics Statement

Institutional Review Board of Wuhan University School of Medicine granted approval for this study. Written informed consent was obtained from all participants.

## Author Contributions

BY, XW and PC designed the study and wrote the research protocol. PC, XL, BY, XW, ZL and JR did the literature review, managed the field survey, quality control, and statistical analysis, and prepared the manuscript draft. XW and BY contributed to the revisions in depth for the manuscript. ZL, XW, BY and JR supervised the survey and checked the data. All authors contributed to and approved the final manuscript.

## Funding

We appreciate the grant support provided by the National Key R&D Program of China (2018YFC1314600), Project of Humanities and Social Sciences of the Ministry of Education in China (20YJCZH204), the National Natural Science Foundation of China (NO.71503192) and Wuhan Health and Family Planning Commission Research Fund (No. WX17B16).

## Conflict of Interest

The authors declare that the research was conducted in the absence of any commercial or financial relationships that could be construed as a potential conflict of interest.
